# Antioxidant and Cytoprotective Effects of *Tibetan Tea* and Its Phenolic Components

**DOI:** 10.3390/molecules23020179

**Published:** 2018-01-24

**Authors:** Hong Xie, Xican Li, Zhenxing Ren, Weimin Qiu, Jianlan Chen, Qian Jiang, Ban Chen, Dongfeng Chen

**Affiliations:** 1 School of Chinese Herbal Medicine, Guangzhou University of Chinese Medicine, Guangzhou 510006, China; xiehongxh1@163.com (H.X.); weiss99@163.com (W.Q.); 15219389939@163.com (J.C.); jiangqiande920711@163.com (Q.J.); 15119635373@163.com (B.C.); 2 Innovative Research & Development Laboratory of TCM, Guangzhou University of Chinese Medicine, Guangzhou 510006, China; Guangzhou 510006, China; 3 School of Basic Medical Science, Guangzhou University of Chinese Medicine, Guangzhou 510006, China; 13192669534@163.com; 4 The Research Center of Basic Integrative Medicine, Guangzhou University of Chinese Medicine, Guangzhou 510006, China

**Keywords:** Kangzhuan, cytoprotection, catechins, steric effect, antioxidant mechanisms

## Abstract

Tibetan tea (Kangzhuan) is an essential beverage of the Tibetan people. In this study, a lyophilized aqueous extract of Tibetan tea (**LATT**) was prepared and analyzed by HPLC. The results suggested that there were at least five phenolic components, including gallic acid, and four catechins (i.e., (+)-catechin, (−)-catechin gallate (**CG**), (−)-epicatechin gallate (**ECG**), and (−)-epigallocatechin gallate). Gallic acid, the four catechins, and **LATT** were then comparatively investigated by four antioxidant assays: ferric reducing antioxidant power, 2-phenyl-4,4,5,5-tetramethylimidazoline-1-oxyl 3-oxide radical (PTIO•) scavenging, 1,1-diphenyl-2-picryl-hydrazl radical scavenging, and 2,2′-azino-bis(3-ethylbenzo-thiazoline-6-sulfonic acid) radical scavenging assays. In these assays, LATT, along with the five phenolic components, increased their antioxidant effects in a concentration-dependent manner; however, the half maximal scavenging concentrations of **ECG** were always lower than those of **CG**. Gallic acid and the four catechins were also suggested to chelate Fe^2+^ based on UV-visible spectral analysis. Ultra-performance liquid chromatography coupled with electrospray ionization quadrupole time-of-flight tandem mass spectrometry (UPLC−ESI−Q−TOF−MS/MS) analysis suggested that, when mixed with PTIO•, the five phenolic components could yield two types of radical adduct formation (RAF) products (i.e., tea phenolic dimers and tea phenolic-PTIO• adducts). In a flow cytometry assay, (+)-catechin and **LATT** was observed to have a cytoprotective effect towards oxidative-stressed bone marrow-derived mesenchymal stem cells. Based on this evidence, we concluded that LATT possesses antioxidative or cytoprotective properties. These effects may mainly be attributed to the presence of phenolic components, including gallic acid and the four catechins. These phenolic components may undergo electron transfer, H^+^-transfer, and Fe^2+^-chelating pathways to exhibit antioxidative or cytoprotective effects. In these effects, two diastereoisomeric CG and ECG showed differences to which a steric effect from the 2-carbon may contribute. Phenolic component decay may cause RAF in the antioxidant process.

## 1. Introduction

Tibetan tea (“Zangcha” in Chinese, Figure S1), a member of the fully fermented tea (dark tea) family, has a history of approximately 1000 years in China. It has become an essential beverage for millions of Tibetan people. The Tibetan Plateau varies in altitude between 3000 and 5000 meters and is not well-suited for cultivating vegetables, fruit, or trees. Thus, Tibetan people eat various meat products, such as beef, milk, and butter tea. These high-protein, high-lipid foods can effectively help them to resist the severe cold of high altitude. However, this diet may increase the risk of cardiovascular and indigestion diseases. 

It has been reported that Tibetan tea can effectively lower blood pressure, remove blood lipids, and reduce the generation of atherosclerosis [1,2]. Intake of Tibetan tea is also hypothesized to blunt the effects of high-altitude hypoxia [3]. This tea is indispensable for Tibetan life and is thus called “Tibetan tea” by the Chinese, even though the tea is actually manufactured in Sichuan (a Han nationality Province in China). The tea is processed through five main working procedures with 30 processing technologies; these technologies were listed as Chinese Intangible Cultural Heritage in 2008 [4]. The whole process takes approximately 6 months, during which some chemical components have possibly degraded and some fungal populations have also been altered [5]. All of these give Tibetan tea a characteristic taste and potential beneficial effects. 

Nevertheless, as a traditional tea with ethnological characteristics, Tibetan tea has received very little interest from scientists. In the PubMed database, only three papers about Tibetan tea can be retrieved from 1991 to 2016 [3,6,7]. 

According to their working procedures and materials, Tibetan tea products are classified into three main types: Kangzhuan, Jinjian, and Kangjian. The most popular type is Kangzhuan, i.e., brick tea. The present study attempted to use Kangzhuan as a representative to estimate the antioxidant or cytoprotective effects of Tibetan tea and examined the possible mechanisms. 

## 2. Results and Discussion

**Figure 1:**
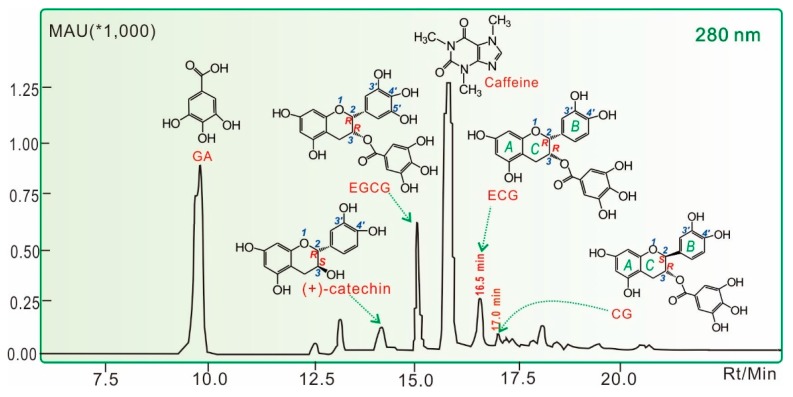
HPLC of **LATT.** **GA**, gallic acid; **CG**, (−)-catechin gallate; **ECG**, (−)-epicatechin gallate; **EGCG**, (−)-epigallocatechin gallate. The determining wavelength was 280 nm; The longitudinal axis was the strength of absorbance. MAU, milli-absorbance unit; Rt, retention time; Min, minute.

In this study, the prepared **LATT** was analyzed by the HPLC method, and at least five phenolic components were found, including gallic acid (**GA**) and four catechins [(+)-catechin and its galloylated derivatives **CG**, **ECG**, and **EGCG**, Figure 1]. The contents of **GA**, (+)-catechin, **EGCG**, **ECG**, and **CG** were calculated as 0.44 ± 0.02 g/100 g, 0.11 ± 0.01 g/100 g, 0.24 ± 0.01 g/100 g, 0.13 ± 0.01 g/100 g, and 0.04 ± 0.01 g/100 g, respectively. Each of their contents in **LATT** was lower than in green tea [8]. The difference was reported to be due to the comprehensive working protocols (especially fermentation [9]) and production seasons [10]. In the present study, their lower levels may also be related to the sample preparation. Our preliminary experiment using distilled water to dissolve LATT found the huge peaks of caffeine and gallic acid to severely interfere with the determination of other peaks. Thus, we had to use methanol to prepare LATT solution for determination. Methanol however cannot fully extract the phenolic components from a lyophilized aqueous extract. This also led to a lower level of caffeine (0.72 ± 0.01 g/100 g).

**Figure 2:**
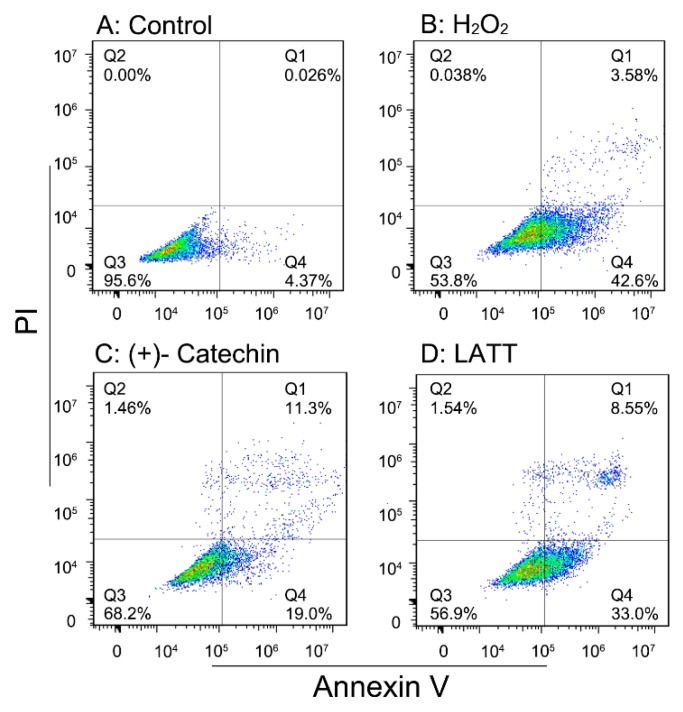
Typical flow cytometry diagram of cytoprotection of (+)-catechin and **LATT** in oxidatively stressed bmMSCs. The assay was conducted to distinguish live cells (**Q3**), necrotic cells (**Q2**), early apoptotic cells (**Q4**), and late apoptotic/necrotic cells (**Q1**).

Our previous study demonstrated that the antioxidant capacity of plants can be mainly attributed to the existence of total phenolics [11]. In the present experiments, five phenolic components and **LATT** were comparatively evaluated using various antioxidant assays, including the ferric reducing antioxidant power (FRAP), PTIO•-scavenging, ABTS^+^•-scavenging, and DPPH•-scavenging assays. The half maximal scavenging concentration (SC_50_) values in these assays revealed that the five phenolic components always exhibited stronger antioxidant abilities than **LATT** itself (Table 1). On the basis of this evidence and previous reports [12,13], it can be assumed that these phenolic components may be responsible for the antioxidant effects of LATT. This is consistent with data from the flow cytometry assay, in which (+)-catechin (68.2%) gave a higher uninjured cell population than LATT (56.9%) (Figure 2). The study only measured (+)-catechin for cytoprotection because (+)-catechin possesses the basic, most typical skeleton (Figure 1), and other galloylated catechins (especially **EGCG**) have been widely studied for their cytoprotection previously [14,15,16,17].

As mentioned in the previous literature, the FRAP assay at pH 3.6 is essentially an electron-transfer (ET) process [18]. The data (Figure S2 and Table 1) showed that the five phenolic components in **LATT** could effectively increase the FRAP percentages, suggesting that these phenolic components may undergo the ET pathway to scavenge free radicals. 

The ET pathway, however, is usually accompanied by an H^+^-transfer pathway [18]. The PTIO•-scavenging assay, an H^+^-transfer-involved reaction [19,20], was hence performed in the study. The five phenolic components and **LATT** dose-dependently increased their PTIO•-scavenging efficacies (Figure S3), indicating that H^+^-transfer may be involved in the antioxidant process.

**Figure 3:**
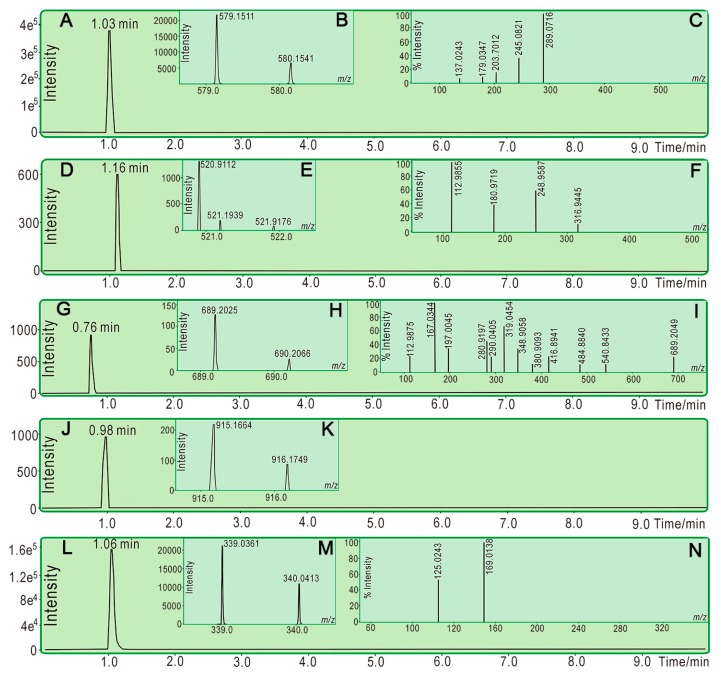
Typical UPLC−ESI−Q−TOF−MS/MS spectra of (+)-catechin (**A**–**F**) and (+)-catechin derivatives (**G**–**K**) for RAF investigation. (**A**) Chromatogram of RAF product of (+)-catechin-(+)-catechin when the formula [C_30_H_28_O_12_-H]^−^ was extracted; (**B**) Primary MS spectra of RAF product of (+)-catechin-(+)-catechin; (**C**) secondary MS spectra of RAF product of (+)-catechin-(+)-catechin; (**D**) chromatogram of RAF product of **C**-PTIO when the formula [C_28_H_30_N_2_O_8_-H]^−^ was extracted; (**E**) primary MS spectra of RAF product of (+)-catechin-PTIO; (**F**) secondary MS spectra of RAF product of (+)-catechin-PTIO; (**G**) chromatogram of RAF product of **EGCG**-PTIO when the formula [C_35_H_34_N_2_O_13_-H]^−^ was extracted; (**H**) primary MS spectra of RAF product of **EGCG**-PTIO; (**I**) secondary MS spectra of RAF product of **EGCG**-PTIO.; (**J**) chromatogram of RAF product of **EGCG**-**EGCG** when the formula [C_44_H_36_O_22_-H]^−^ was extracted; (**K**) primary MS spectra of RAF product of **EGCG**-**EGCG**. **EGCG** for the reference compound of (+)-catechin derivative; (**L**) chromatogram of RAF product of **GA**-**GA** when the formula [C_14_H_12_O_10_-H]^−^ was extracted; (**M**) primary MS spectra of RAF product of **GA**-**GA**; (**N**) secondary MS spectra of RAF product of **GA**-**GA**. The other spectra are listed in 6–8.

It is worth mentioning that, after the ET and H^+^-transfer reaction, phenolic antioxidants may be transferred into free radicals. The phenolic antioxidant radical may form a covalent adduct with another free radical in the decay process. This is called a radical adduct formation (RAF) reaction [21]. As shown in Figure 3A,B, the reaction product of PTIO• with (+)-catechin gave a peak with *m*/*z* 579.2 at 1.03 min in the ultra-performance liquid chromatography coupled with electrospray ionization quadrupole time-of-flight tandem mass spectrometry (UPLC−ESI−Q−TOF−MS/MS) analysis. The *m*/*z* value (580) of the product was exactly double the molecular weight of (+)-catechin, and its secondary MS resembled (+)-catechin itself (Figure 3C), suggesting a dimerization reaction in the RAF pathway. In addition, (+)-catechin can react with PTIO• to give (+)-catechin-PTIO (*m*/*z* 520.9, Figure 3D–F). 

As a galloylated derivative of (+)-catechin, **EGCG** was also found to yield a relevant **EGCG**-PTIO product (*m*/*z* 689.2, Figure 3G–I) and dimeric **EGCG**-**EGCG** (*m*/*z* 915.2, Figure 3J–K). In addition to **EGCG**, other galloylated derivatives (**CG** and **ECG**) similarly gave RAF products (Figures S6 and S7). **GA** also gave a dimer of **GA**-**GA**. The dimer was identified by a peak with *m*/*z* 339.0, which is exactly double the molecular weight of **GA** in primary MS spectra, which yielded secondary MS spectra similar to **GA** itself, i.e., *m*/*z* 125, 170 (Figure 3L–N and Figure S8). Taken together, **GA** and the four catechins could generate RAF products when mixing with PTIO• radicals. It can be deduced that **GA** and the four catechins undergo the RAF pathway to exert their antioxidant actions. 

The above RAF results are generally consistent with previous findings from ABTS•^+^- and DPPH•-scavenging assays. ABTS•^+^- and DPPH•-scavenging actions have been shown to have multiple antioxidant pathways involved in hydrogen atom transfer (HAT) [22], ET [23,24], H^+^-transfer [25], sequential electron proton transfer [23,24,26], or RAF [27,28,29]. In the present study, the phenolic components in **LATT** effectively scavenged ABTS•^+^ and DPPH• radicals (Table 1 and Figures S4 and S5). This further suggests that the phenolic components in **LATT** can undergo ET, H^+^-transfer, and RAF to display antioxidant (reactive oxygen species-scavenging) effects. 

**Figure 4:**
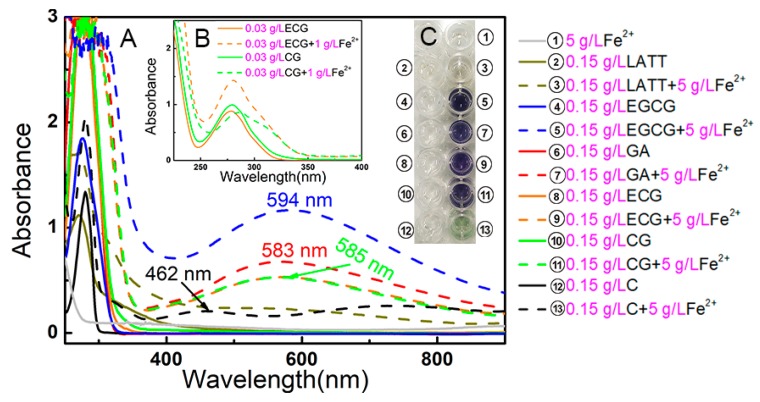
(**A**) UV spectra of Fe^2+^-chelating complex; (**B**) UV-visible spectra of **ECG** and **CG** in Fe^2+^-chelating reaction; (**C**) Appearance of solutions.

Reactive oxygen species generation is usually linked to transition metals (especially Fe^2+^ and Cu^+^). For instance, Fe^2+^ can catalyze H_2_O_2_ to form •OH radicals via the Fenton reaction (Fe^2+^ + H_2_O_2_ → Fe^3+^ + •OH + OH^−^) [26]. Thus, chelation of Fe^2+^ in solution can greatly reduce the catalytic potential to inhibit •OH generation [23,30]. In the present study, the five phenolic components and **LATT** exhibited Fe^2+^-chelating abilities. As shown in Figure 4A,B, GA bound Fe^2+^ to generate a blue solution with λ_max_ 583 nm absorption. The **EGCG**-Fe^2+^ complex produced a dark-blue solution with λ*_max_* 594 nm. Each of the **CG**-Fe^2+^ and **ECG**-Fe^2+^ complexes gave a navy solution with λ_max_ 585 nm. The (+)-catechin-Fe^2+^ complex yielded a green solution with λ_max_ = 462 nm. These data imply that all five phenolic compounds and **LATT** exhibit Fe^2+^-chelating abilities and that Fe^2+^-chelation may play a role in the antioxidative process of **LATT** [31].

**Figure 5:**
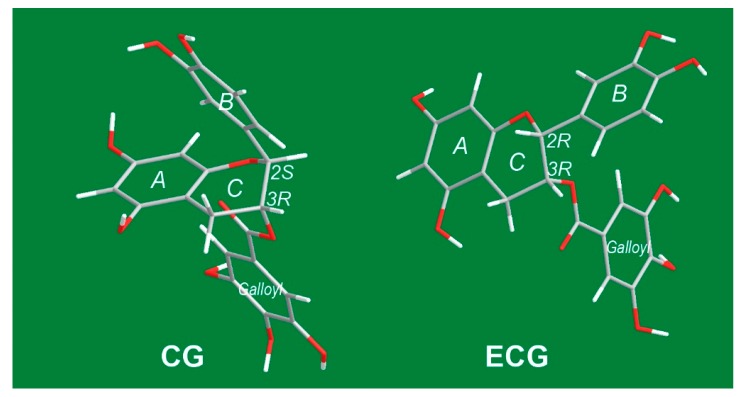
Stick-models of preferential conformations of **CG** and **ECG** (Red is for O atom; White is H and gray is C. Double bonds are not shown.).

**Figure 6:**
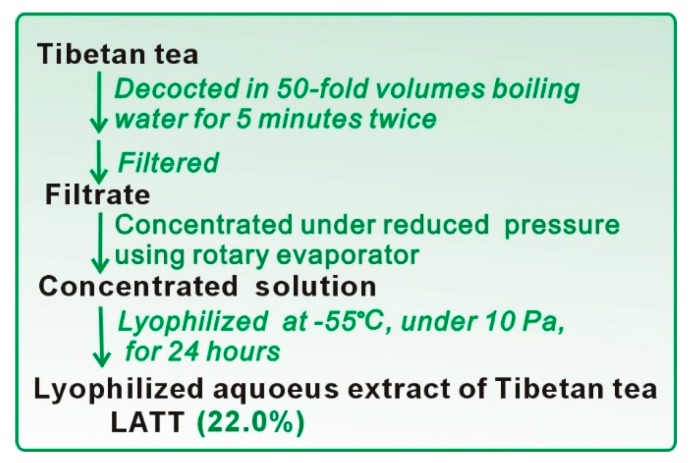
Preparation of **LATT**.

It must be emphasized that **CG** and **ECG** are two diastereoisomers: **CG** and **ECG** take (***2S***, ***3R***) and (***2R***, ***3R***) absolute configurations, respectively. Thus, the sole difference between **CG** and **ECG** is the absolute steric configuration at the 2-carbon (Figure 1 and Figure 6). This difference gives them distinctive molecular shapes. In the **CG** molecule, the *B* ring and galloyl moiety are arranged on two different sides, while they appear on the same side in **ECG** (Figure 5). Thus, an unbalanced array of chemical moieties enhances the polarity of **ECG**, and **ECG** (16.5 min) is eluted ahead of **CG** (17.0 min) in reversed-phase column HPLC (Figure 1). More importantly, such different steric configurations can also affect antioxidant abilities, including radical-scavenging and Fe^2+^-chelation. As shown in Table 1, **ECG** always exhibited higher antioxidant levels than **CG** in DPPH•-scavenging, ABTS^+^•-scavenging, PTIO•-scavenging, and FRAP assays. This may be because two very large ipsilateral moieties (*B* ring and galloyl moiety) may lead to repulsion to increase molecular energy and ET potential. This presumption is consistent with previous findings that *cis-*catechins exhibit a higher antioxidant ability than their epimers [32,33]. 

In Fe^2+^-chelation, there is also a difference between **CG** and **ECG**. As illustrated in Figure 4B, **ECG**-Fe^2+^ gave stronger UV absorbance than ECG itself, while **CG**-Fe^2+^ exhibited weaker UV absorbance than **CG** itself. This difference can be explained by the absolute configurational difference at the 2-carbon. As two moieties with metal chelation potential [34,35], the *B* ring and the galloyl residue, are on the same side in **ECG**, this configuration can promote their synergistic binding to Fe^2+^. This synergistic effect, however, cannot exist in **CG**, because they are separated distantly (Figure 5).

In a word, there are substantial differences between **CG** and **ECG** in terms of molecular polarity, radical scavenging, and Fe^2+^-chelation. This stereochemistry may help us to further understand differences between catechins and corresponding “epi”-catechins [36,37].

## 3. Materials and Methods

### 3.1. Animals and Chemicals

Tibetan tea (Kangzhuan, Lot No. 20120427) was purchased from Sichuan Ya’an Tea Factory Co., Ltd. (Sichuan, China). Sprague-Dawley (SD) rats (4 weeks) were obtained from the Animal Center of Guangzhou University of Chinese Medicine. (+)-Catechin (C_15_H_14_O_6_, M.W. 290.27, CAS 154-23-4, 98%, Figure S9) was purchased from Shanghai Aladdin Chemistry Co., Ltd. (Shanghai, China). (−)-Epicatechin gallate (**ECG**, C_22_H_18_O_10_, M.W. 442.37, CAS 1257-08-5, 98%, Figure S9) and (−)-epigallocatechin gallate (**EGCG**, C_22_H_18_O_11,_ M.W. 458.37, CAS 989-51-5, 98%, Figure S9) were purchased from Sichuan Weikeqi Biological Technology Co., Ltd. (Chengdu, China). (−)-Catechin gallate (**CG**, C_22_H_18_O_10_, M.W. 442.37, CAS 130405-40-2, 98%, Figure S9) and gallic acid (**GA**, C_7_H_6_O_5_, M.W. 170.12, CAS 149-91-7, 98%, Figure S9) were obtained from Chengdu PureChem-Standard Co., Ltd. (Chengdu, China). Caffeine (CAS 58-08-2, 98%) was from the Guangdong Guanghua Chemical Factory Co., Ltd. (Shantou, China). DPPH• (1,1-diphenyl-2-picryl-hydrazl radical), Trolox (± -6-hydroxyl-2,5,7,8-tetramethlychromane-2-carboxylic acid), and 2,4,6-tripyridyl triazine (TPTZ) were purchased from Sigma-Aldrich Shanghai Trading Co. (Shanghai, China). PTIO• (2-phenyl-4,4,5,5-tetramethylimidazoline-1-oxyl-3-oxide radical) was from TCI Chemical Co. (Shanghai, China). (NH_4_)_2_ABTS [2,2′-azino-bis(3-ethylbenzo-thiazoline-6-sulfonic acid) diammonium salt] was obtained from the Amresco Chemical Co. (Solon, OH, USA). Low-glucose Dulbecco’s modified Eagle’s medium (L-DMEM), fetal bovine serum (FBS), and trypsin were purchased from Gibco (Grand Island, NY, USA). An annexin V/propidium iodide (PI) assay kit was purchased from Abcam (Cambridge, UK). Methanol and water were of HPLC grade. All other reagents used in this study were purchased as analytical grade from the Guangzhou Chemical Reagent Factory (Guangzhou, China). 

### 3.2. Preparation of Lyophilized Aqueous Extract of Tibetan Tea (LATT)

Tibetan tea was extracted based on the guidance of the manufacturer. In brief, the newly-purchased Tibetan tea (Kangzhuan) was decocted twice using 50-fold distilled water at 100 °C for 5 min. The extract was concentrated and lyophilized to prepare a lyophilized aqueous extract of Tibetan tea (**LATT**, Figure S10 and Figure 6). **LATT** was stored at 4 °C for further exploration. 

### 3.3. HPLC Analysis for Phenolic Components in LATT

The **LATT** methanolic solution (48.6 mg/mL) was analyzed using an HPLC method. The HPLC analysis was performed on a Shimadzu LC-20A (Shimadzu Co., Kyoto, Japan) equipped with an Agilent 5 TC-C_18_ (250 mm × 4.6 mm, 5 μm) (Agilent Technologies Inc., Palo Alto, CA, USA). The mobile phase consisted of A (methanol) and B (0.5% formic acid in water) with gradient elution: 0–5 min (90–70% B), 5–9 min (70–50% B), 9–13 min (50–40% B), 13–20 min (maintain 40% B), 20–22 min (40–90% B), and 22–25 min (remain 90% B). The flow rate was 0.8 mL/min, injection volume was 20 μL, column temperature was 35 °C, and absorption was measured at 280 nm. In the study, **GA**, (+)-catechin, **EGCG**, **ECG**, **CG**, and caffeine were identified by comparing their retention times with those of authentic samples. 

### 3.4. Ferric Reducing Antioxidant Power (FRAP) Assay

The FRAP assay was carried out based on Benzie and Strain’s method [36]. Briefly, the FRAP reagent was prepared freshly by mixing 10 mM TPTZ, 20 mM FeCl_3_, and 0.25 M acetate buffer (pH 3.6) at 1:1:10. The test sample (*x* = 2–10 μL) was added to (20 − *x*) μL of 95% ethanol followed by 80 μL of FRAP reagent. After incubation for 30 min, the mixture was measured for the A_593nm_ value at ambient temperature using distilled water as the blank. The relative reducing antioxidant power of the sample compared to the maximum absorbance was calculated by the following formula:

$$Relative\;reducing\;effect\;\%=\frac{A-A_{\min}}{A_{\max}-A_{\min}}\times 100\%$$

where *A*_min_ is the lowest *A*_593nm_ value in the experiment, *A* is the *A*_593nm_ value of the reaction mixture with sample, and *A*_max_ is the greatest *A*_593nm_ value in the experiment. 

### 3.5. Free Radical-Scavenging Assays In Vitro

The free radical-scavenging assays included the PTIO•-scavenging assay, DPPH•-scavenging assay, and ABTS^+^•-scavenging assay. The PTIO•-scavenging assay was based on our method [22]. In brief, 80 μL of aqueous PTIO• solution (0.1 mM) was mixed with 20 μL of an aqueous or alcoholic solution of sample at various concentrations. The mixture was incubated at 50 °C for 30 min, and the absorbance at 560 nm was measured using a microplate reader (Multiskan FC, Thermo Scientific, Shanghai, China). The PTIO•-scavenging сapacity % was calculated as:

$$Scavenging\;capacity\;\%=\frac{A_{0}-A}{A_{0}}\times 100\%$$

where *A*_0_ is the absorbance of the control without sample and *A* is the absorbance of the reaction mixture with sample. 

The ABTS^+^•-scavenging assay was based on a previous report [37]. ABTS^+^• was produced by mixing 200 μL of (NH_4_)_2_ABTS (7.4 mM) with 200 μL of K_2_S_2_O_8_ (2.6 mM). After incubation in the dark for 12 h, the mixture was diluted with methanol (approximately 1:20) so that the absorbance at 734 nm was 0.30 ± 0.01. Then, the diluted ABTS^+^• solution (80 μL) was added to 20 μL of an ethanolic (or aqueous) solution of sample at various concentrations and mixed thoroughly. After the reaction mixture stood for 3 min, the absorbance at 734 nm was measured using a microplate reader. The ABTS^+^•-scavenging сapacity % was calculated using the formula described in the PTIO•-scavenging assay.

The DPPH•-scavenging assay was based on previous reports [38]. In the assay, 80 μL of an ethanolic solution of DPPH• (0.1 mM) was mixed with 20 μL of an ethanolic (or aqueous) solution of sample at various concentrations. After incubation for 30 min, the mixture was measured for absorbance at 519 nm. The DPPH•-scavenging сapacity % was calculated using the formula described in the PTIO•-scavenging assay. SC_50_ was defined as the antioxidant concentration to give a scavenging capacity % half the value of that in the absence of antioxidant.

### 3.6. UPLC−ESI−Q−TOF−MS/MS Analysis of Reaction Products of PTIO• with Phenolic Components

UPLC−ESI−Q−TOF−MS/MS spectra of reaction products of PTIO• with phenolic components were determined according to the method described by our report [39]. The methanolic solutions of phenolic components were mixed with a solution of PTIO• radicals in methanol at a molar ratio of 1:2, and the resulting mixtures were incubated for 24 h at room temperature. The product mixtures were filtered through a 0.22-μm filter and measured using a UPLC-ESI-Q-TOF-MS/MS system equipped with a C_18_ column (2.0 mm i.d. × 100 mm, 2.2 μm, Shimadzu Co., Kyoto, Japan). The mobile phase was used for elution and consisted of a mixture of methanol (phase A) and water (phase B). The column was eluted at a flow rate of 0.3 mL/min with the following gradient elution program: 0–10 min, 60–100% A; 10–15 min, 100% A. The sample injection volume was set at 1 μL to separate the components, column temperature was 40 °C; Q-TOF-MS/MS analysis was conducted with a Triple TOF 5600 *^plus^* mass spectrometer (AB SCIEX, Framingham, MA, USA) equipped with an ESI source, which was run in the negative ionization mode. The scan range was set at 100–2000 Da. The system was run with the following parameters: ion spray voltage, −4500 V; ion source heater, 550 °C; curtain gas (CUR, N_2_), 30 psi; nebulizing gas (GS1, air), 50 psi; Tis gas (GS2, air), 50 psi. The declustering potential (DP) was set at −100 V, whereas the collision energy (CE) was set at −40 V with a collision energy spread (CES) of 20 V. The RAF products were quantified by extracting the corresponding formula (e.g., [C_28_H_30_N_2_O_8_-H]^−^ for (+)-catechin-PTIO• and [C_30_H_28_O_12_-H]^−^ for (+)-catechin dimer) from the total ion chromatogram and integrating the corresponding peak.

### 3.7. Cytoprotective Effect Assay 

The MSC culture was performed in terms of our previous reports with slight modifications [14,40]. Briefly, bone marrow was obtained from the femur and tibia of rats. The marrow samples were diluted with L-DMEM containing 10% FBS. MSCs were prepared by gradient centrifugation at 900× *g* for 30 min on 1.073 g/mL Percoll. The prepared cells were transferred into culture flasks at a density of 1 × 10^4^/cm^2^. The MSCs at passage 3 were estimated for cultured cell homogeneity using detection of CD44 by flow cytometry and used for the investigation. Injured MSCs were prepared by treatment with H_2_O_2_ (200 μM). After incubation for 3 h, MSCs in the control group and H_2_O_2_ group were incubated for 24 h in DMEM, while MSCs in the sample groups were incubated for 24 h in DMEM with (+)-catechin and **LATT**. 

After culturing, cells were assayed using flow cytometry [41]. Briefly, the cultured cells were harvested with trypsin (0.05%) digestion in phosphate-buffered saline (PBS). The cells (5 × 10^5^) were collected by centrifugation and then resuspended in 500 μL of binding buffer. At the same time, 5 μL of annexin V-FITC and 5 μL of propidium iodide were added. After incubation at room temperature for 5 min in the dark, fluorescence was measured by flow cytometry (Accuri C6, Franklin Lakes, BD, USA) with standard software.

### 3.8. UV Spectral Determination of Fe^2+^-Chelation 

UV spectra for the Fe^2+^-chelation of **LATT** and its phenolic components were determined according to the method described by our report [42,43,44]. Briefly, 75 μL of a methanolic solution of sample (1 mg/mL) and 25 μL of an aqueous solution of FeCl_2_•4H_2_O (100 mg/mL) were added to 400 μL of an aqueous mixture of distilled water and methanol (1:1). The solution was mixed vigorously. The resulting mixture was incubated at room temperature for 1 h. Subsequently, the product mixture was collected and a spectrum was obtained using a UV-visible spectrophotometer (Unico UV 2600A, Unico Co., Shanghai, China) from 200 to 900 nm. Next, 200 μL of the supernatant was transferred to a 96-well plate and photographed using a smartphone. 

### 3.9. Statistical Analysis

The SC_50_ values were obtained by linear regression analysis. All linear regression analysis was conducted with Origin 6.0 professional software. Measurement of significant differences between the mean SC_50_ values of the sample and positive controls was performed using one-way ANOVA or the T-test. The analysis was performed using SPSS software 13.0 (SPSS Inc., Chicago, IL, USA) for Windows. *p* < 0.05 was considered to be significant. 

## 4. Conclusions

Tibetan tea comprises at least five phenolic components, i.e., **GA** and four catechins [(+)-catechin, **CG**, **ECG**, and **EGCG**]. The antioxidant or cytoprotective effects of **LATT** may mainly be attributed to the existence of **GA,** four catechins, and other phenolic components. Among the four catechins, (+)-catechin was demonstrated to protect bmMSCs from oxidative stress-induced apoptosis. **GA** and the four catechins may undergo ET, H^+^-transfer, and Fe^2+^-chelating pathways to exhibit antioxidative or cytoprotective effects. In these effects, diastereoisomeric **CG** and **ECG** were different, and the difference can be attributed to that of a steric effect at the 2-carbon. Phenolic component decay may give rise to RAF products in the antioxidant process. 

## Figures and Tables

**Table 1:** The FRAP and SC_50_ values of **LATT, ECG, EGCG, CG, C, GA**, and Trolox.

	FRAP	PTIO•-Scavenging	ABTS^+^•-Scavenging	DPPH•-Scavenging
**LATT** μg/mL	33.2 ± 1.2	531.9 ± 26.0	15.0 ± 0.4	48.7 ± 1.7
**ECG** μg/mL(μM)	2.7 ± 0.1 (6.0 ± 0.2 ^a^)	23.1 ± 0.6 (52.2 ± 1.4 ^a^)	0.8 ± 0.0 (1.8 ± 0.0 ^a^)	2.5 ± 0.1 (5.7 ± 0.2 ^a^)
**EGCG** μg/mL(μM)	4.9 ± 0.2 (10.9 ± 0.5 ^b^)	23.8 ± 0.23 (53.7 ± 0.5 ^a^)	1.4 ± 0.1 (3.0 ± 0.1 ^b^)	2.9 ± 0.1 (6.9 ± 0.3 ^b^)
**CG** μg/mL(μM)	5.3 ± 0.1 (11.9 ± 0.1 ^b^)	28.2 ± 3.1 (63.8 ± 7.1 ^b^)	1.3 ± 0.0 (2.9 ± 0.1 ^b^)	4.1 ± 0.2 (9.4 ± 0.4 ^b^)
(+)-catechin μg/mL(μM)	10.8 ± 0.5 (37.3 ± 1.9 ^d^)	71.2 ± 4.0 (161.0 ± 9.1 ^d^)	1.1 ± 0.0 (3.6 ± 0.1 ^c^)	5.1 ± 0.1 (18.2 ± 0.3 ^d^)
**GA** μg/mL(μM)	3.1 ± 0.1 (18.1 ± 0.8 ^c^)	50.4 ± 0.64 (113.9 ± 1.4 ^c^)	0.7 ± 0.0 (4.3 ± 0.2 ^d^)	2.1 ± 0.1 (12.6 ± 0.4 ^c^)
Trolox μg/mL(μM)	11.2 ± 0.2 (44.6 ± 1.0 ^e^)	23.9 ± 0.7 (54.1 ± 1.5 ^a^)	5.4 ± 1.2 (21.4 ± 4.9 ^f^)	9.2 ± 1.1 (36.3 ± 4.5 ^e^)

SC_50_ values are expressed as the mean ± SD (*n* = 3). Mean values with different letters (a, b, c, d, e, or f) in same column are significantly different (*p* < 0.05). All dose–response curves are detailed in Figure 2, Figure 3, Figure 4 and Figure 5.

## Supplementary Materials

The following are available online. Figure S1: The photo of Tibetan tea. Figure S2: (A) The dose–response curves of catechins and positive controls from FRAP assay. (B) The dose–response curves of LATT from FRAP assay. Figure S3: (A) The dose–response curves of catechins and Trolox in PTIO• scavenging. (B) The dose–response curves of LATT in PTIO• scavenging. Figure S4: (A) The dose–response curves of catechins and positive controls in ABTS^+^•-scavenging. (B) The dose–response curves of LATT in ABTS^+^•-scavenging. Figure S5: (A) The dose–response curves of catechins and positive controls in DPPH•-scavenging. (B) The dose–response curves of LATT in DPPH•-scavenging. Figure S6: (A) Chromatogram of RAF product of (−)-epigallocatechin gallate-(−)-epigallocatechin gallate when formula [C_44_H_36_O_20_-H]^−^ was extracted. (B) Primary MS spectra of RAF product of (−)-epigallocatechin gallate-(−)-epigallocatechin gallate. (C) Secondary MS spectra of RAF product of (−)-epigallocatechin gallate-(−)-epigallocatechin gallate. Figure S7: (A) Chromatogram of RAF product of (−)-catechin gallate-(−)-catechin gallate when formula [C_44_H_36_O_20_-H]^−^ was extracted. (B) Primary MS spectra of RAF product of (−)-catechin gallate-(−)-catechin gallate. (C) Secondary MS spectra of RAF product of (−)-catechin gallate-(−)-catechin gallate. Figure S8: Secondary MS spectra of gallic acid. Figure S9: Certificates of analysis of gallic acid, (+)-catechin, (−)-catechin gallate (CG), (−)-epicatechin gallate (ECG), and (−)-epigallocatechin gallate (EGCG). Figure S10: The appearance of the lyophilized aqueous extract of Tibetan tea.

## Abbreviations

The following abbreviations are used in this manuscript:

ABTS	[2,2′-azino-bis(3-ethylbenzo-thiazoline-6-sulfonic acid diammonium salt)]
bmMSCs	bone marrow-derived mesenchymal stem cells
CG	(−)-catechin gallate
DMEM	Dulbecco’s modified Eagle’s medium
DPPH•	1,1-diphenyl-2-picryl-hydrazl
ET	electron transfer
ECG	(−)-epicatechin gallate
EGCG	(−)-epigallocatechin gallate
FBS	fetal bovine serum
FRAP	ferric reducing antioxidant power
GA	gallic acid
HAT	hydrogen atom transfer
LATT	lyophilized aqueous extract of *Tibetan tea*
PTIO•	2-phenyl-4,4,5,5-tetramethylimidazoline-1-oxyl 3-oxide radical
ROS	reactive oxygen species
RAF	radical adduct formation
SD	standard deviation
TPTZ	2,4,6-tris(2-pyridyl-s-triazine)
