# Supplementary material for: Antioxidant and Cytoprotective Effects of Tibetan Tea and Its Phenolic Components
Source: Molecules. 2018 Jan 24;23(2):179. doi: 10.3390/molecules23020179 (PMC6017439; doi:10.3390/molecules23020179)
Supplement: Supplementary file 1 [file molecules-23-00179-s001.pdf]

Supplemental Materials

Antioxidant and Cytoprotective Effects of *Tibetan Tea* and its Phenolic Components

Hong Xie [1, 2, †], Xican Li [1, 2, *, †], Zhenxing Ren [3, 4], Weimin Qiu [1], Jianlan Chen [1], Qian Jiang [1, 2], Ban Chen[1, 2] and Dongfeng Chen [3, 4, *]

[1] School of Chinese Herbal Medicine, Guangzhou University of Chinese Medicine, Guangzhou 510006, China; xiehongxh1@163.com (H.X.); lixican@126. com(X.L.); 657216262@qq.com (W.Q.); 790992174@qq.com(J.C.); jiangqiande920711@163.com (Q.J.) ; 1002363510@qq.com (B.C.)

[2] Innovative Research & Development Laboratory of TCM, Guangzhou 510006, China;

[3] School of Basic Medical Science, Guangzhou University of Chinese Medicine, Guangzhou 510006, China; 251351061@qq.com (Z.R.)

[4] The Research Center of Basic Integrative Medicine, Guangzhou University of Chinese Medicine, Guangzhou 510006, China.

*Corresponding author. E-mail: lixican@126. com(X.L.); chen888@gzucm.edu.cn (D.C.). **Tel**: +86-20-39358076

†Equal contributors

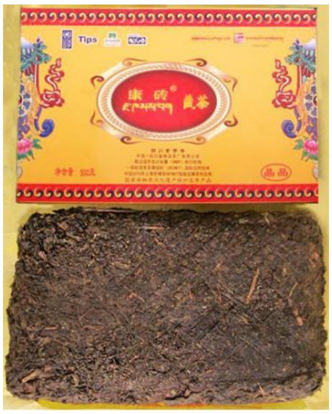

Figure S1**:** The photo of Tibetan tea (Kangzhuan).

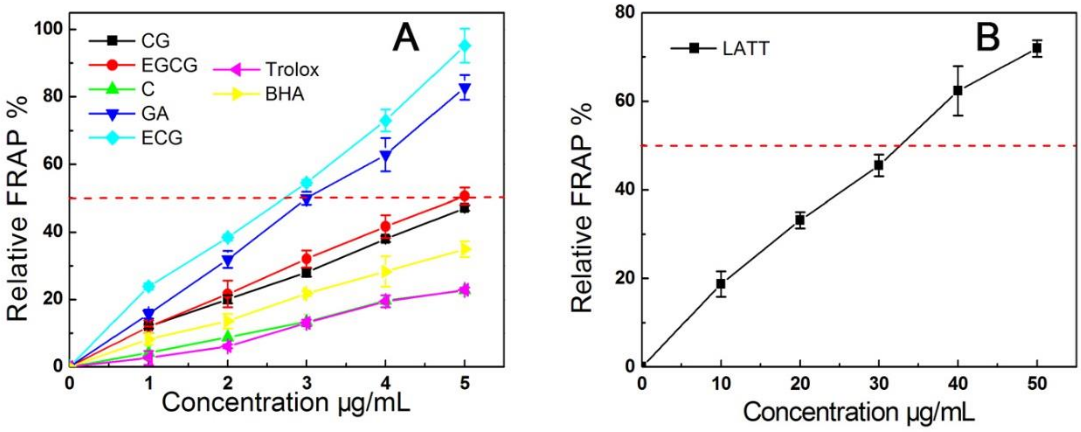

Figure S2 (A) The dose response curves of catechins and Trolox in FRAP assay. (B) The dose response curves of LATT in FRAP assay. Each value is expressed as mean ± SD (*n* = 3).

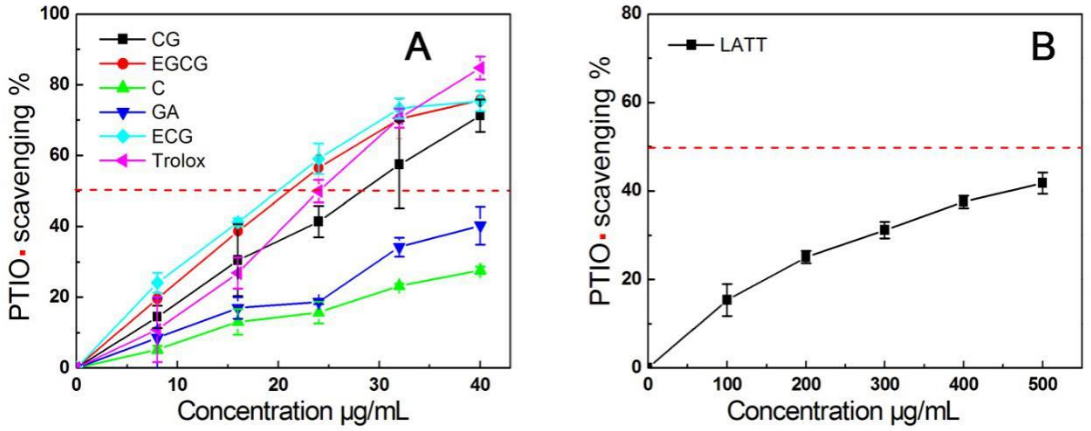

Figure S3. (A) The dose response curves of catechins and Trolox in PTIO• scavenging. (B) The dose response curves of LATT in PTIO• scavenging. Each value is expressed as mean ± SD (*n* = 3).

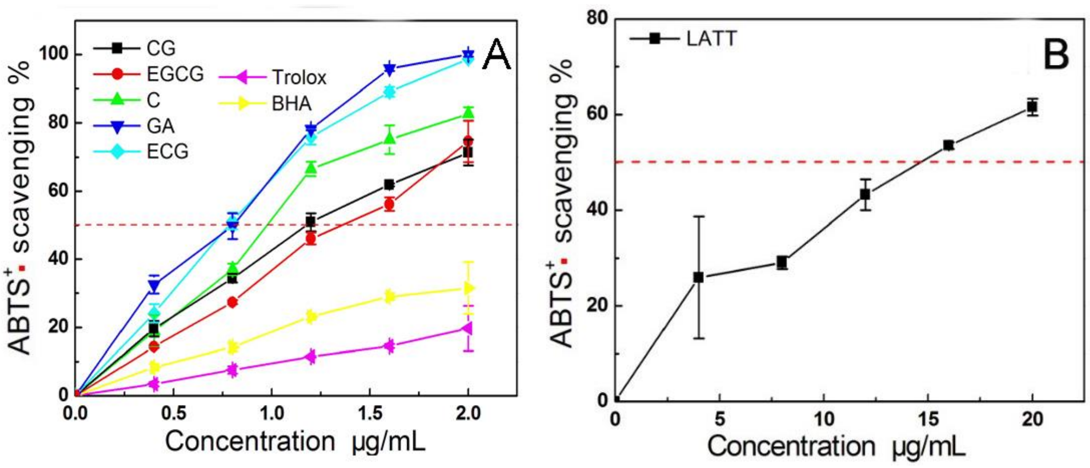

Figure S4. (A) The dose response curves of catechins and Trolox in $ABTS^{+}$• scavenging. (B) The dose response curves of LATT in $ABTS^{+}$• scavenging. Each value is expressed as mean ± SD (*n* = 3).

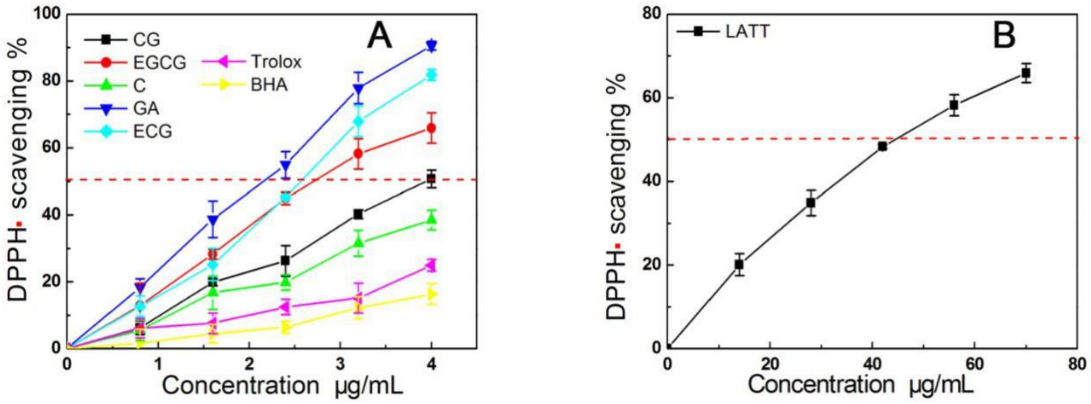

Figure S5. (A) The dose response curves of catechins and Trolox in DPPH• scavenging. (B) The dose response curves of LATT in DPPH• scavenging. Each value is expressed as mean ± SD (*n* = 3).

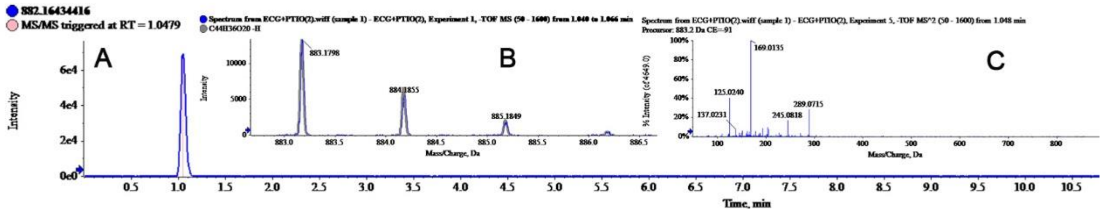

Figure S6. (A) Chromatogram of RAF product of (-)-epigallocatechin gallate-(-)-epigallocatechin gallate when formula $[C_{44}H_{36}O_{20}\text{-}H]^-$ was extracted. (B) Primary MS spectra of RAF product of (-)-epigallocatechin gallate-(-)-epigallocatechin gallate. (C) Secondary MS spectra of RAF product of (-)-epigallocatechin gallate-(-)-epigallocatechin gallate.

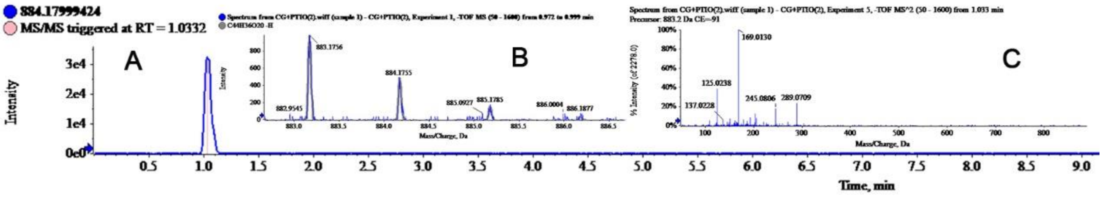

Figure S7. (A) Chromatogram of RAF product of (-)-catechin gallate-(-)-catechin gallate when formula $[C_{44}H_{36}O_{20}\text{-}H]^-$ was extracted. (B) Primary MS spectra of RAF product of (-)-catechin gallate-(-)-catechin gallate. (C) Secondary MS spectra of RAF product of (-)-catechin gallate-(-)-catechin gallate.

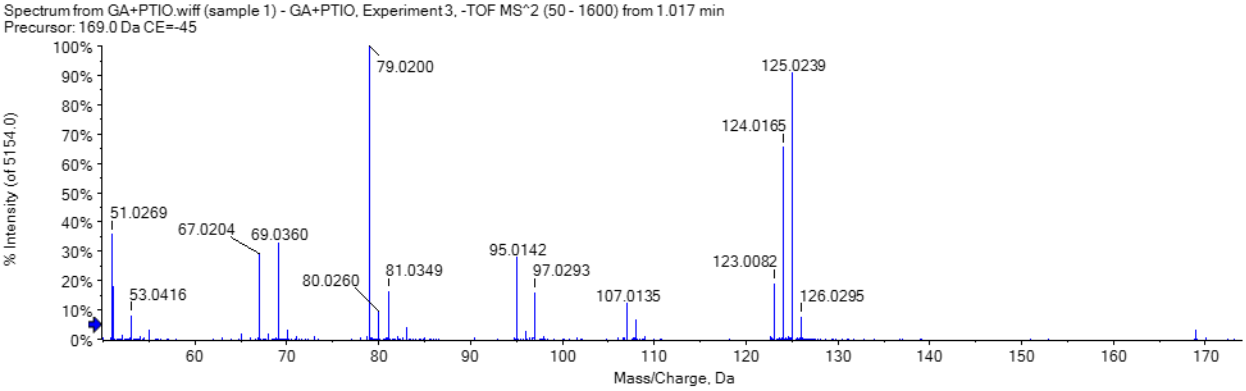

Figure S8. Secondary MS spectra of gallic acid.

Figure S9. Certificates of analysis of gallic acid, (+)-catechin, (-)-catechin gallate (CG), (-)-epicatechin gallate (ECG), and (-)-epigallocatechin gallate (EGCG).

(The subsequent 10 pages)

产品分析证书
Certificate of Analysis

中文名称：没食子酸

English Name：Gallic acid
别名(Alias): 3,4,5-Trihydroxybenzoic acid

产品编码(Cat. No.):BP0608
CAS Number: 149-91-7
分子式(M. F.): $C_7H_6O_5$
分子量(M. W.): 170.12

批号(Batch No.): PRF7091942
报告日期(Report date): 2016/9/19

Structure

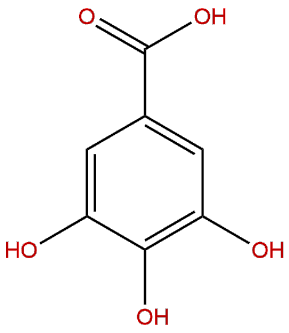

检验结果（Analytical result）：

**检验项目（Test Item）**	**检验指标（Specifications）**	**检验结果（Results）**
外观Appearance	Type of white needle crystal	Type of white needle crystal
干燥失重Loss on drying	＜3.0%	1.31%
纯度Purity (HPLC-DAD,270nm)*	≥98.0%	99.96%
质谱Mass	170.12±1	Conforms
核磁NMR	Comply with the structure	Conforms

*** 色谱图见附件（Please find HPLC chromatography attached.）**

贮存条件（Storage）:2~8℃

复测期（Retest date）:two years (2018-09-18) under conditions list above.

备注（Remarks）：如遇质量问题，请于收到产品之日起 15 日内与我们联系。

In case of quality issue, please contact us within 15 days after receipt of the product.

QC: Zhang Ling

Date: 2016年9月19日

QA: Wu Qi

Date: 2016年9月19日

Tel：+86-28-82633987 Fax：+86-28-82633165
http://www.biopurify.com Email：sales@biopurify.com biopurify@gmail.com

SAMPLE INFORMATION

Sample Name:	Gallic acid PRF7091942	Acquired By:	System
Sample Type:	Unknown	Sample Set Name:	
Vial:	27	Acq. Method Set:	Gallic acid
Injection #:	1	Processing Method:	Samples
Injection Volume:	10.00 ul	Channel Name:	270.0nm
Run Time:	25.0 Minutes	Proc. Chnl. Descr.:	PDA 270.0 nm
Date Acquired:	2016-9-19 10:59:13 CST		
Date Processed:	2016-9-19 12:48:38 CST		

Auto-Scaled Chromatogram

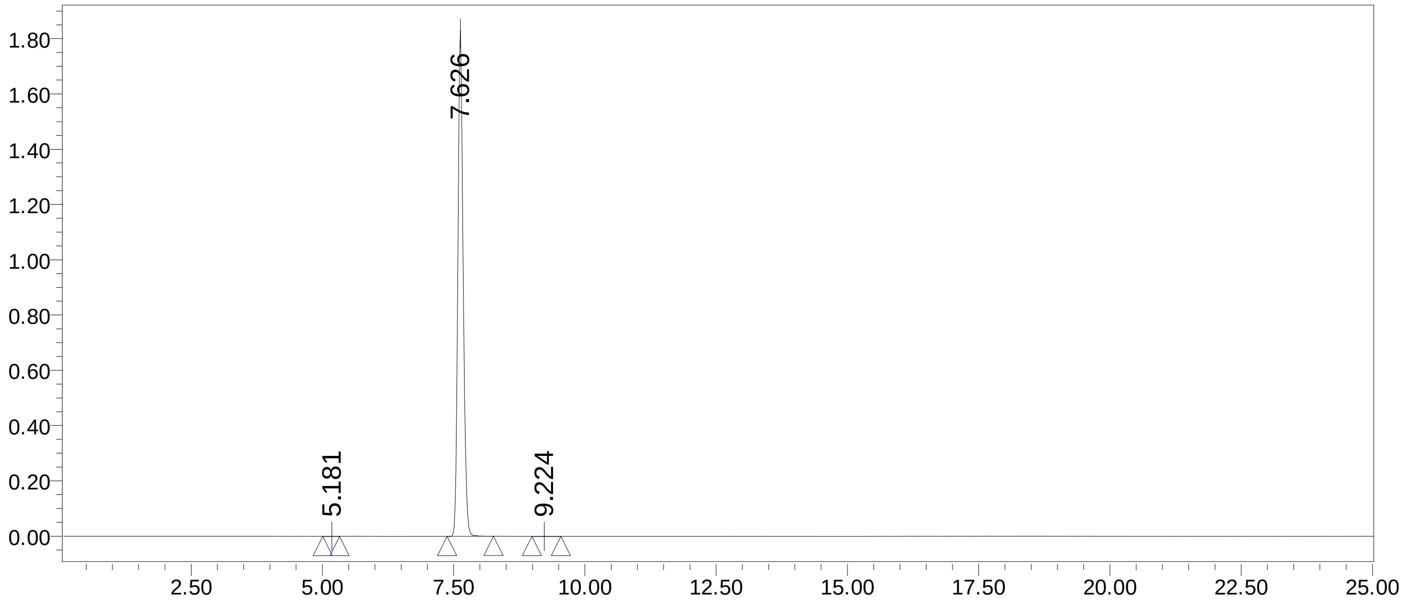

Peak Results

	RT	Area	% Area	USP Plate Count	USP Resolution
1	5.181	2434	0.02	20612.47	
2	7.626	12916674	99.96	26396.43	14.23
3	9.224	2242	0.02	30185.08	7.55

Suite 601, Shanda Building, No. 196 xinjinqiao Road, Pudong District, Shanghai 201206, China
TELEPHONE : 400 620 6333 FAX : +86-21 5102 6000 / +86-21 5032 3701
TECH EMAIL : tech@aladdin-e.com

CERTIFICATE OF ANALYSIS

PRODUCT NAME:
(+)-Catechin,analytical standard,≥97%

**ITEM NUMBER:**	C114051
**LOT NUMBER:**	B1707017
BRAND:	Aladdin
CAS NUMBER:	154-23-4,225937-10-0
MDL NUMBER:	MFCD00075649
FORMULA:	C15H14O6
FORMULA WEIGHT:	290.27
QUALITY RELEASE DATE:	2017-02-10 13:59:46

TEST	SPECIFICATION MIX. MAX.	VALUE	RESULT
Appearance	orange to brown powder or crystals		Consistent
Proton NMR spectrum	Conforms to Structure		Conforms to Structure
Purity(HPLC)	98 % 100 %	100 %	99.084200 %
Water by Karl Fischer	0 % 1 %	1 %	0.638000 %

Julian
ALADDIN REAGENT
Fengxian,Shanghai,CN

Aladdin warrants, that at the time of the quality release or subsequent retest date this product conformed to the information contained in this publication. The current Specification sheet may be available at www.aladdin-e.com. For further inquiries, please contact Technical Service. Purchaser must determine the suitability of the product for its particular use. See reverse side of invoice or packing slip for additional terms and conditions of sale

VERSION NUMBER: 1

四川省维克奇生物科技有限公司
Sichuan Weikeqi Biological Technology Co., Ltd.

质检报告

Quality Test Report

产品名称	表没食子儿茶素没食子酸酯	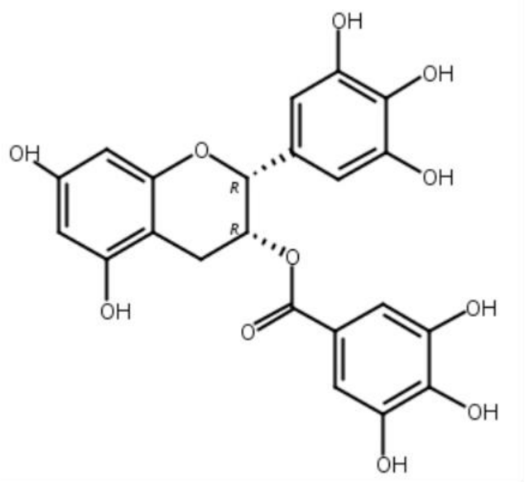
Product Name	(-)-Epigallocatechin gallate	
数量	12.93g	
Amount	12.93g	
批号	wkq16062503	
Batch Number	wkq16062503	
报告日期	2016.06.25	
Report Date	2016.06.25	
生产日期	2016.06.12	
Manufacture Date	2016.06.12	

分子式	C22H18011	分子量	458.37	CAS 号	989-51-5

项目	规定	结果
Characters	Provisions	Results
性状	白色粉末	符合
Characteristics	White powder	Complies
纯度 Purity(HPLC)	≥98%	99.57%
Analyst:	Checker:	Q.C.director
检验员:	复核员:	负责人

中文网站：www.weikeqi-biotech.com Official Web: http://www.weikeqi-biological.com
邮箱：scwkqzj@scdzp.com International Mail: scwkqlql@scdzp.com
Tel：0086-028-81700200 4008005713 Fax：0086-028-81705658
Office Address: Room 3,20th floor,Jinsha Lansheng, Middle Qingjiang Road No. 63,Qingyang District, Chengdu, Sichuan.
Lab Address：8th floor, Chengdu Institute of Biology, Chinese Academy of Sciences

表没食子儿茶素没食子酸酯液相图谱

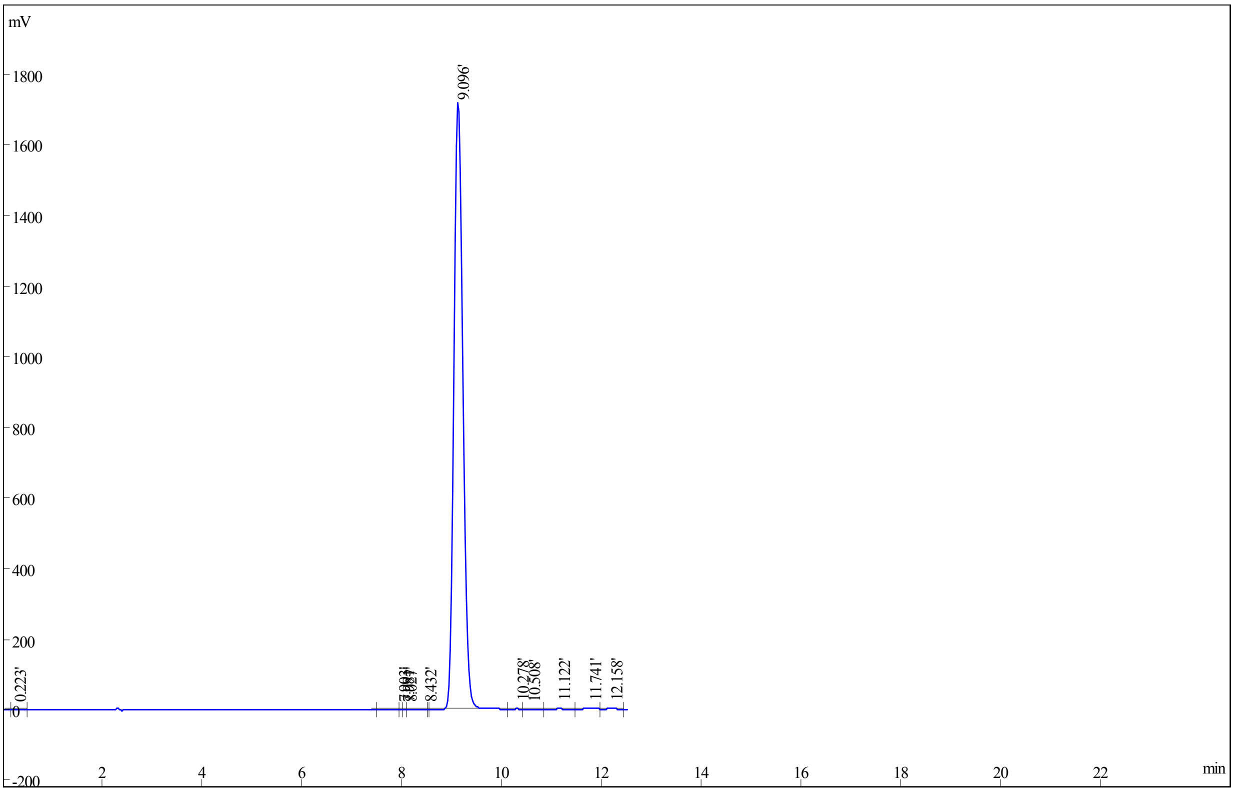

序号	保留时间	名称	浓度	峰面积
1	0.223		0.0005411	117
2	7.903		0.002319	503
3	7.981		0.0006399	139
4	8.027		0.0002648	57
5	8.432		0.002173	471
6	9.096		99.57	21602987
7	10.278		0.08244	17887
8	10.508		0.07987	17329
9	11.122		0.08403	18231
10	11.741		0.1459	31667
11	12.158		0.03859	8373
总计			100	21697761

质检报告

Quality Test Report

产品名称	表儿茶素没食子酸酯	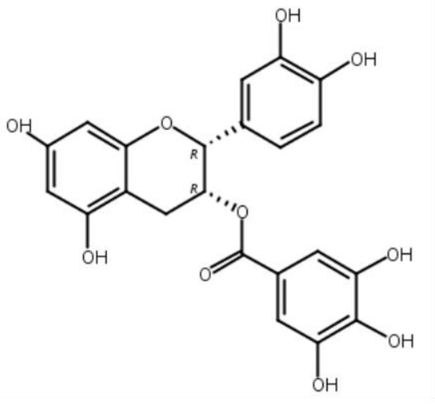
Product Name	(-)-Epicatechin gallate	
数量	12.25g	
Amount	12.25g	
批号	wkq16071505	
Batch Number	wkq16071505	
报告日期	2016.07.15	
Report Date	2016.07.15	
生产日期	2016.07.02	
Manufacture Date	2016.07.02	

分子式	C22H18010	分子量	442.37	CAS 号	1257-08-5

项目	规定	结果
Characters	Provisions	Results
性状	白色粉末	符合
Characteristics	White powder	Complies
纯度 Purity(HPLC)	≥98%	98.06%
Analyst:	Checker:	Q.C.director
检验员:	复核员:	负责人

中文网站：www.weikeqi-biotech.com　　Official Web: http://www.weikeqi-biological.com
邮箱：scwkqzj@scdzp.com　　International Mail: scwkqlql@scdzp.com
Tel：0086-028-81700200　　4008005713　　Fax：0086-028-81705658
Office Address: Room 3,20th floor,Jinsha Lansheng, Middle Qingjiang Road No. 63,Qingyang District, Chengdu, Sichuan.
Lab Address：8th floor, Chengdu Institute of Biology, Chinese Academy of Sciences

表儿茶素没食子酸酯液相图谱

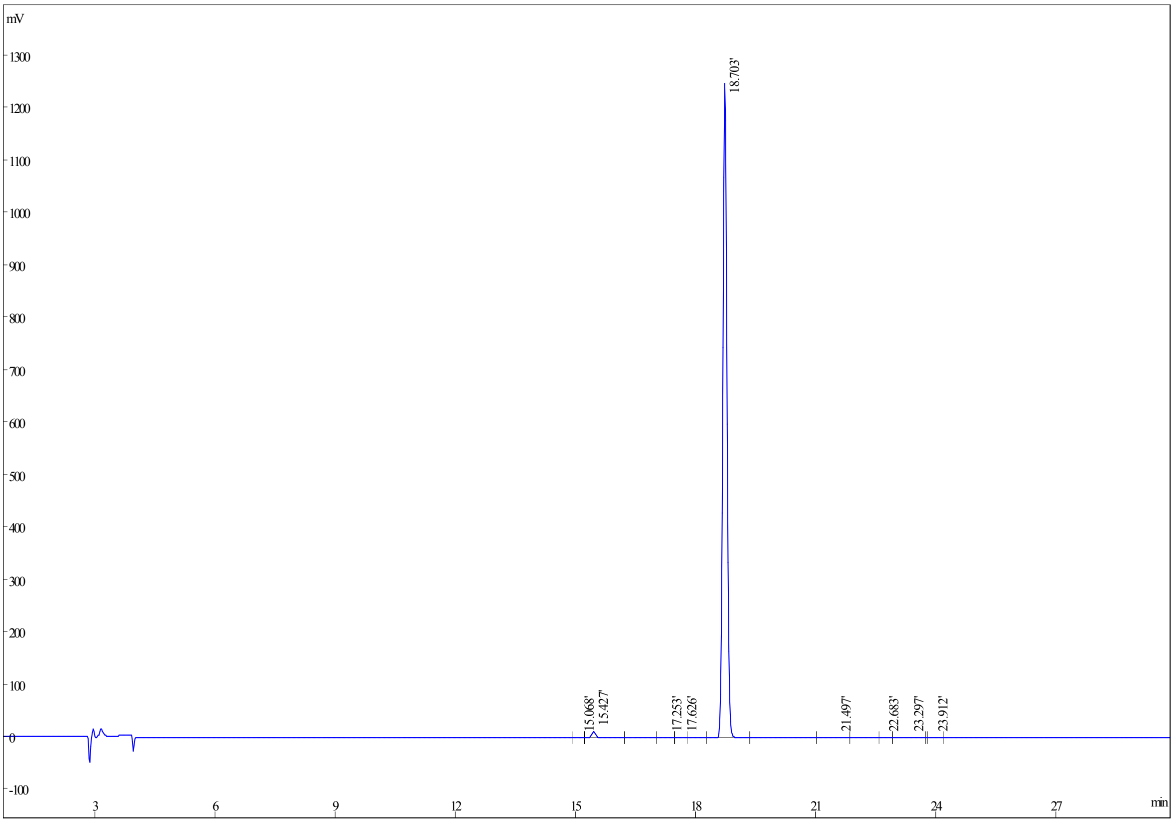

序号	保留时间	浓度	峰面积	峰高	理论塔板数	有效塔板数	峰拖尾因子
1	15.068	0.1815	16607	2581	127965	0	1.12
2	15.427	1.27	116196	12883	68265	37	1.88
3	17.253	0.0593	5425	429	43426	697	1.18
4	17.626	0.1666	15246	2217	153300	3230	0.98
5	18.703	98.06	8971982	1267076	162789	6149	1.05
6	21.497	0.12	10978	915	74912	6701	2.17
7	22.683	0.03452	3158	466	261474	29475	1.23
8	23.297	0.04846	4433	229	33795	4217	0.62
9	23.912	0.05533	5062	679	240094	32845	0.93
总计		100	9149087	1287475			

中文网站：www.weikeqi-biotech.com　Official Web: http://www.weikeqi-biological.com
邮箱：scwkqzj@scdzp.com　International Mail: scwkqlql@scdzp.com
Tel：0086-028-81700200　4008005713　Fax：0086-028-81705658
Office Address: Room 3,20th floor,Jinsha Lansheng, Middle Qingjiang Road No. 63,Qingyang District, Chengdu, Sichuan.
Lab Address：8th floor, Chengdu Institute of Biology, Chinese Academy of Sciences

产品分析证书
Certificate of Analysis

中文名称：儿茶素没食子酸酯

English Name: Catechin gallate
别名(Alias): (-)-Catechin gallate

产品编码(Cat. No.): BP0323
CAS Number: 130405-40-2
分子式(M. F.): $C_{22}H_{18}O_{10}$
分子量(M. W.): 442.376

批号(Batch No.): PRF7080501
报告日期(Report date): 2016/8/5

Structure

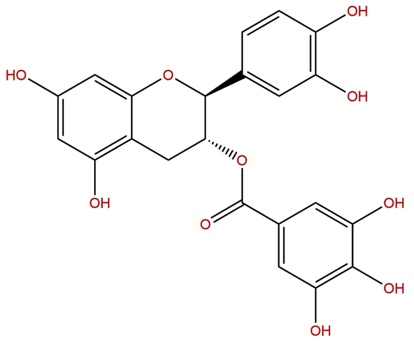

检验结果（Analytical result）：

检验项目（Test Item）	检验指标（Specifications）	检验结果（Results）
外观Appearance	Rice yellow powder	Rice yellow powder
干燥失重Loss on drying	＜3.0%	1.6%
纯度Purity (HPLC-DAD, 275nm)*	≥98.0%	98.45%
质谱Mass	442.376±1	Conforms
核磁NMR	Comply with the structure	Conforms

* 色谱图见附件（Please find HPLC chromatography attached.）

贮存条件（Storage）:2~8℃
复测期（Retest date）:two years (2018-08-04) under conditions list above.
备注（Remarks）：如遇质量问题，请于收到产品之日起 15 日内与我们联系。
In case of quality issue, please contact us within 15 days after receipt of the product.

QC: Zhang Ling　　　　QA: Wu Qi
Date: 2016年8月5日　　　　Date: 2016年8月5日

Chengdu Biopurify Phytochemicals Ltd. 成都普瑞法科技开发有限公司 质检专用章 Quality Approval Seal

Tel：+86-28-82633987　Fax：+86-28-82633165
http://www.biopurify.com　Email：sales@biopurify.com　biopurify@gmail.com

SAMPLE INFORMATION

Sample Name:	CG PRF7080501	Acquired By:	System
Sample Type:	Unknown	Sample Set Name:	
Vial:	64	Acq. Method Set:	CG
Injection #:	1	Processing Method:	Samples
Injection Volume:	10.00 ul	Channel Name:	275.0nm
Run Time:	25.0 Minutes	Proc. Chnl. Descr.:	PDA 275.0 nm
Date Acquired:	2016-8-5 12:19:40 CST		
Date Processed:	2016-8-5 13:01:33 CST		

Auto-Scaled Chromatogram

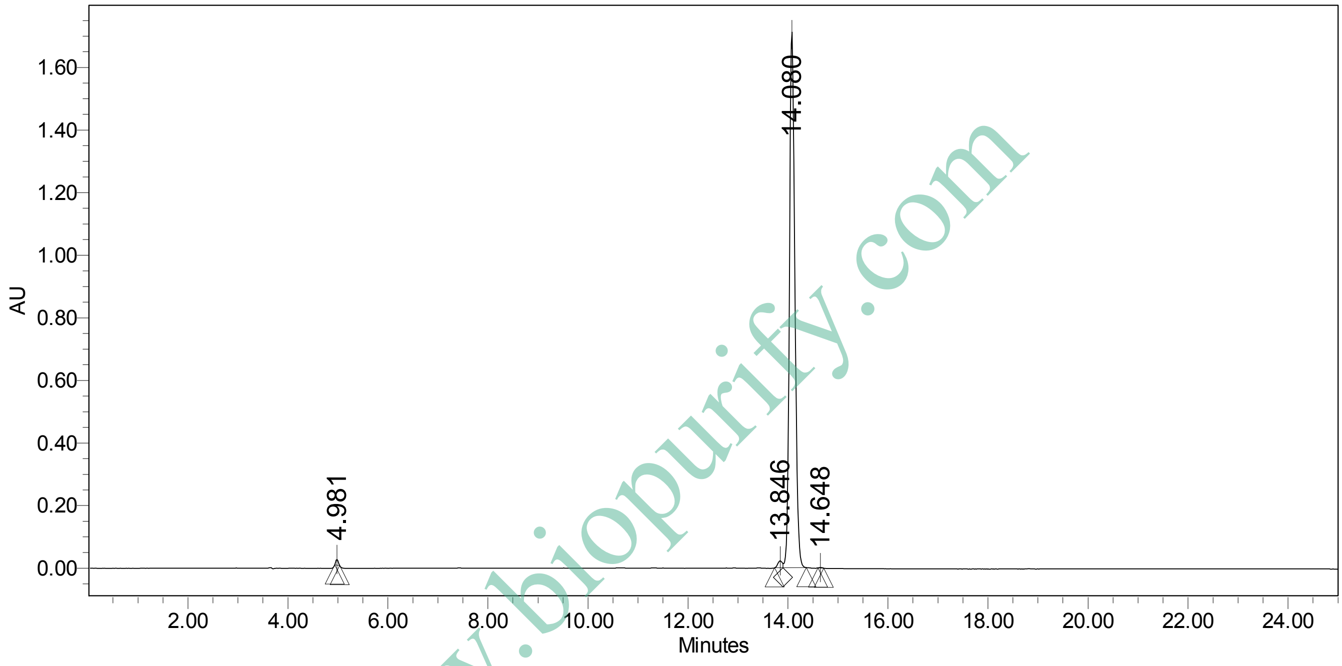

www.biopurify.com

Peak Results

	Name	RT	Area	% Area	USP Plate Count	USP Resolution
1		4.981	63900	0.49	39606.30	
2		13.846	133607	1.02		89.20
3		14.080	12946053	98.45	78783.73	1.55
4		14.648	6083	0.05	202606.32	3.48

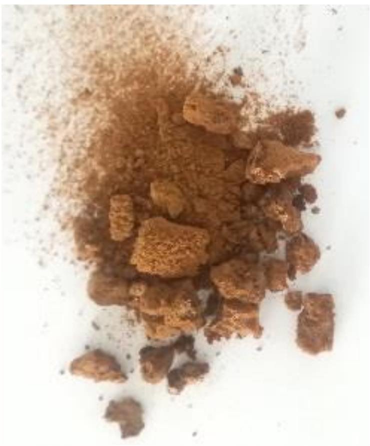

Figure S10: The appearance of the lyophilized aqueous extract of Tibetan tea (LATT).
